# Health Survey in a Peruvian health system (ENSSA): design, methodology and general results

**DOI:** 10.11606/S1518-8787.2019053001135

**Published:** 2019-03-26

**Authors:** Renán Quispe Llanos, Rofilia Ramírez Ramírez, Martha Tizón Palacios, Claudio Flores Flores, Alfredo Borda-Olivas, Roger Araujo Castillo, Juan Guanira, Risof Solis Condor, Manuel Catacora Villasante, Yamilée Hurtado-Roca

**Affiliations:** I Gerencia de Gestión de la Información. EsSalud. Lima, Perú; II Instituto de Evaluación de Tecnologías en Salud e Investigación – IETSI. EsSalud. Lima, Perú; III Oficina de Inteligencia e Información Sanitaria. EsSalud. Lima, Perú; IV Universidad Peruana de Ciencias Aplicadas. Escuela de Medicina. Lima, Perú; V Universidad Nacional Mayor de San Marcos. Lima, Perú

**Keywords:** Health Surveys, methods, Socioeconomic Survey, methods, Sampling Studies, Health Systems, Encuestas Epidemiológicas, métodos, Encuesta Socioeconómica, métodos, Muestreo, Sistemas de Salud

## Abstract

**OBJECTIVE:**

To report the design, methodology and initial results of the National Socioeconomic Survey of Access to Health of the EsSalud Insured.

**RESULTS:**

There were interviews in 25,000 homes, surveying 79,874 people, of which 62,659 were affiliated to EsSalud. The insured people are mainly males (50.6%) with a higher technical education level (39.7%). The insured population has mostly independent (95.0%) and own (68.1%) home. Only 34.5% of the insured practice some sport or physical exercise; 14.0% of the population suffers from a chronic disease; 3.5% have diabetes; and 7.1%, arterial hypertension. In the last three months, 35.4% of the members needed medical attention; of these, only 73.1% received health care and the remaining 10.9% were treated in pharmacies or non-formal health care services.

**RESULTS:**

The 25,000 homes were interviewed, surveying 79,874 people, of which 62,659 were affiliated to EsSalud. The insured people are mainly males (50.6%) with a higher technical education level (39.7%). The insured population has mostly independent (95.0%) and own (68.1%) home. Only 34.5% of the insured practice some sport or physical exercise; 14.0% of the population suffers from a chronic disease; 3.5% have diabetes; and 7.1%, arterial hypertension. In the last three months, 35.4% of the members needed medical attention; of these, only 73.1% received health care and the remaining 10.9% were treated in pharmacies or non-formal health care services.

**CONCLUSIONS:**

This survey is the first performed in the population of EsSalud affiliates, applied at the national level, and has socio-economic and demographic data of the insured, their distribution, risk factors of health, prevalence of health problems and the degree of access to health services.

## RESUMEN

**OBJETIVO:**

Reportar el diseño, metodología y resultados iniciales de la Encuesta Nacional Socioeconómica de Acceso a la Salud de los Asegurados de EsSalud.

**MÉTODOS:**

La Encuesta se ejecutó en los 24 departamentos del país. Los temas investigados fueron: características de la vivienda y miembros del hogar, educación, salud, empleo e ingreso y gastos del hogar. Se realizó un tipo de muestreo bi-etápico: la unidad primaria de muestreo estuvo conformada por conglomerados compuestos por una manzana dentro del ámbito de cobertura del centro asistencial; la unidad secundaria de muestreo fueron viviendas particulares donde habitaba al menos un asegurado a EsSalud. Se analizaron los datos de 62,659 afiliados y se muestran porcentajes ajustados por el factor de expansión. Para las comparaciones, se utilizó la prueba de chi-cuadrado.

**RESULTADOS:**

Se entrevistaron 25000 viviendas, encuestándose a 79,874 personas, de las cuales 62,659 eran afiliados a EsSalud. Los afiliados son principalmente varones (50.6%) con un nivel de educación técnico superior (39.7%). La población afiliada cuenta mayoritariamente con vivienda independiente (95.0%) y propia (68.1%). Solo el 34.5% de los asegurados practica algún deporte o ejercicio físico. El 14.0% de la población padece de alguna enfermedad crónica; el 3.5% presenta diabetes; y el 7.1%, hipertensión arterial. En los últimos tres meses, el 35.4% de los afiliados necesitaron atención médica; de estos, solo el 73.1% recibieron atención sanitaria y el 10.9% restante se atendieron en farmacias o servicios no formales de atención en salud.

**CONCLUSIONES:**

Esta encuesta es la primera realizada en la población de afiliados a EsSalud, aplicada a nivel nacional, y cuenta con datos socioeconómicos y demográficos de los asegurados, su distribución, factores de riesgo de la salud, prevalencia de los problemas de salud y el grado de acceso a los servicios de salud.

## INTRODUCTION

Health at population level is influenced by several complex and related factors. The model proposed by Omran ^1^ attributes changes in health to the epidemiological transition fundamentally to the demographic, social and economic dynamics of a population. Additionally, population growth is influenced by certain determinants such as population distribution, urbanization and industrialization ^2^ . From this perspective, at the level of public health, it is important to obtain information about the population, its habits, its economic and social dynamics, as well as its environment, in order to establish an analysis of their needs and access to health services. This information needs to be obtained with methods and structured tools in a way that validly allows to extrapolate the findings related to health and its determinants in the population of interest ^3^ ^,^ ^4^ . Therefore, methodologies that allow inference to the general population are required, using the survey technique as a research method that allows obtaining data efficiently and quickly ^5^ .

In Peru, the National Demographic and Family Health Survey (ENDES) ^6^ is carried out periodically, an important source of data that allows obtaining information for public policies. However, given that in Peru there is a public health system differentiated according to the provider (Ministry of Health: MINSA and Social Security: EsSalud), our health system (EsSalud) requires having its own population information assured to establish sanitary policies and decision making that fit both hospital data and population data and generate interventions or measures that lead to the benefit of this population ^7^ . This is how, in 2015, the ENSSA survey was conducted in families with people assigned to the Social Security of Peru, to obtain information at population level and generate evidence for decision making. Some of these results have been published as an input for the decision makers and managers of the institution^8–10^ however, the data collected through this survey could potentially be useful for the scientific community and allow the generation of analytical studies. Consequently, the objective of this article is to describe the detail of the design and methodology aspects of this survey. Additionally, we present results on socioeconomic variables, access to health, lifestyles, accidents and health conditions in relation to life cycle phases and geographic regions of the country.

## METHODS

The National Socioeconomic and Health Access Survey of the EsSalud Insured (ENSSA) was carried out in the 24 departments of the country. The main topics investigated were: sociodemographic characteristics of household members, characteristics of the home and household, health status, employment and income, household expenses, knowledge of the services provided by EsSalud, perception of quality and level of satisfaction. The primary objective of the ENSSA survey was to establish the socioeconomic and demographic characteristics of the insured persons, their distribution, health risk factors, prevalence of health problems and the degree of access.

### Design of the ENSSA Survey

The universe was constituted by all the population insured by EsSalud (owners and their beneficiaries) who resided in private homes occupied throughout the territory of Peru. This definition excluded the population that lived in collective housing (hospitals, prisons, convents, shelters, among others). Because the insured population is a group of the population connected by networks, and, also, because the population by network is a group connected by health care center, sample frames were formed for each of them. The sampling frames had geographical boundaries of the coverage areas of each health center and the information of the Housing and Population Census of 2007 ^11^ . By combining both resources, the urban blocks that formed the coverage area by health care center were identified. The levels of inference covered by the sample correspond to the national level, care network and healthcare center. The Primary Sampling Unit (PSU) was represented by the conglomerate conformed by an area within the scope of coverage of the Health Center. The Secondary Sampling Unit (USM) was the private home where at least one insured person lived in EsSalud. The units of information or analysis were the people that make up the house, surveying all the members that live in it.

To obtain the sample size, given that there are no previous studies that allow knowing the proportion of access to health of the EsSalud insured at Healthcare Center level, an expected value of 0.5 proportion was used. The Assistance Centers were stratified according to the number of people assigned, and, according to the stratum, a level of precision was assigned. Therefore, using a confidence level of 0.95, an expected margin of less than 12% and a design effect of 1.2, the sample size at the national level was 24,640 homes. The selection of the sample was in two stages: in the first stage, the conglomerates (blocks) belonging to the coverage of an Assistance Center whose well-defined geographical area and whose size was expressed in terms of number of dwellings were selected; in the second, occupied private dwellings were selected, existing within the conglomerates where an EsSalud insured person lived in. The selection of the first stage units was carried out randomly and with a probability of selection proportional to a size measure, by the number of dwellings that have it at the Housing and Population Census of 2007. For the selection of the second stage units (dwellings within each conglomerate) it was asked in the households of the block (starting from the northeast point) if some EsSalud insured person lives in them. If the answer was affirmative, the home was selected. The work in the PSU ended with the successful interviewing of four households.

### ENSSA Survey Data Collection

The Survey was carried out using a structured questionnaire and through a direct interview. The information gathering work began on February 1^st^, 2015 and ended on March 31^st^ of the same year. The instrument used for the data collection was a questionnaire applied to households that consisted of 12 sections and a total of 290 questions, the same one that was designed along with officials from the different areas of EsSalud. The content responded to institutional information needs for the decision-making. The variables collected in the survey were self-reported; only in the case of the variable circumference of the abdominal perimeter, the interviewees older than 12 years old were measured; pregnant women, puerperal women and postpartum women of up to 60 days were not included.

The survey takers made a registry of houses in the block assigned to them. This registry included all the dwellings they visited while they inquired about the presence of EsSalud insured people living there. The registration ended when the interviewer got his fourth interview to a home where an EsSalud insured person lived in. Each local supervisor verified the filter sheet to confirm that the record was correct. That is, the survey taker had not omitted any housing and the record reflected the reality of the selected block. The supervision process was permanent throughout the survey and was directly and through re-interviews. Direct supervision consisted in the following of the supervisor with the survey taker at the time of the interview, in order to observe the performance and compliance with the methodology. Likewise, the local supervisors carried out re-interviews with 10% of the production of each interviewer, in order to control the quality of the information gathering.

To validate the survey, a pilot study was carried out with 12 people who formed two work brigades; each brigade was composed of one supervisor and five survey takers. The sample size for the pilot study was 60 dwellings; each survey taker was assigned a district, and within the district a randomly selected block; Within each block the four interviews were developed. The basic condition for conducting the survey was that at least one EsSalud insured person would live there; for this, the interviewer used a filter questionnaire. Once the block was located, the survey taker began his journey through the northwest corner, with the first dwelling; if that dwelling met the established conditions, the survey was taken, otherwise, the adjoining dwelling was continued until its workload was completed. Once the field work was completed, the interviewer reported to the brigade leader and delivered the completed questionnaires for later verification. The time of application of the survey on average was 1 hour and 30 minutes. The coverage was 100% of what was planned. Regarding the omissions, the main one is referred to the distribution of the income of the dependents: it was found that more than 70% does not answer the disaggregated amounts; they only indicate total and net amount.

The interviews with lack of information were classified into two types: 1) Rejection, if one or more members of the household refused to participate. 2) Absenteeism, if one or more members of the household were away from home during the interview. This condition was maintained after the interviewer had visited up to three times the home looking for the person and their local supervisor had verified (a fourth visit) that the person was absent.

### Statistical Analysis

For this initial report of results, the data of 62,659 affiliates interviewed in the ENSSA 2015 survey were analyzed. Continuous variables (age) or discrete variables (income, number of household members) were grouped into smaller categories for better interpretation. To show the results of this first report, they were categorized according to age group and region of the country. The demographic, economic characteristics, access to basic services, type of insured and establishment of inscription, lifestyles (physical activity and eating habits), work accidents and health situation of the members according to type of insured (owner or rightful owner) and type of insurance (pensioner or non-pensioner), using the absolute frequencies and percentages. For the variable of origin and place of birth, they were categorized into geographical areas according to the population distribution made by the National Institute of Statistics and Informatics ^12^ . In all cases, the percentages were calculated adjusted for the expansion factor. All comparisons were made using the chi-square test. The data were processed using the statistical program SPSS version 24.0.

### Expansion Factor

For the estimates derived from the National Socioeconomic Survey and Access to Health of the EsSalud insured to be representative of the total of insured persons, it was necessary to determine the adjustment factor (fraction of number of insured persons among the number of dwellings where the insured lives, under the regime within the coverage area of the healthcare center). With this information, the expansion factor (fraction between the number of insured and the number of dwellings visited where an insured lived within the coverage area of the healthcare center) was established to determine the estimates.

## RESULTS

The coverage of the field work was over 99% in all departments, not being able to complete 20 surveys in the group of non-agricultural insured people and 20 surveys of agricultural insured people. That is to say, it was possible to interview 24,620 dwellings with non-agrarian insured people and 440 homes with agrarian insured people.

A total of 79,874 people were surveyed, of whom 62,659 were affiliated to EsSalud. The percentage of male and female members is similar in each of the age groups: 69.4% of the insured population reside on the coast of the country, most of them concentrated in Lima and Callao (47.4%) and only 5.6% reside in the country’s rainforest. The majority of the insured population is between 18 and 59 years old (55.7%), and only 13% is over 60 years old. Of members between three and five years, 28.5% have not yet begun school, 59.6% of members between 18 and 59 years old have technical or university education and 35.6% have only primary education. The educational level of the population of members aged 60 or more is characterized by being little variable: 37.1% have secondary education, 33.1% have university or technical education and 25.5% have primary education. 48.3% of the insured population over the age of 18 are married, 21.8% are cohabiting and 19.6% are single ( Table 1 ).

**Table 1:** Sociodemographic characteristics of the insured population according to age groups.

Variable	Total		Age groups									

			0–5		6–11		12–17		18–59		≥ 60	
					
	n	%^a^	n	%^a^	n	%^a^	n	%^a^	n	%^a^	n	%^a^
Insured people	62,659	100.0	6,047	10.1	6,444	11.1	5,846	10.1	31,163	55.7	13,159	13.0
Gender												
Male	30,144	50.6	3,114	51.7	3,299	51.8	2,979	51.0	14,266	50.5	6,486	48.7
Female	32,515	49.4	2,933	48.3	3,145	48.2	2,867	49.0	16,897	49.5	6,673	51.3
Birth place												
Lima and Callao^b^	10,900	37.1	1,287	46.7	1,226	44.0	977	40.6	5,110	35.5	2,300	27.7
Costa	16,373	23.7	1,613	23.2	1,634	23.1	1,527	24.4	7,796	23.2	3,803	25.6
Mountain	29,150	32.5	2,368	23.3	2,781	26.0	2,661	28.6	15,084	34.1	6,256	41.7
Rainforest	6,154	6.5	773	6.6	786	6.5	674	6.2	3,146	7.0	775	4.7
Foreigner	82	0.2	6	0.2	17	0.4	7	0.2	27	0.2	25	0.3
Place of residence												
Lima and Callao^b^	14,328	47.4	1,233	47.0	1,201	44.7	967	43.5	6,526	47.0	4,401	54.7
Costa	16,499	22.0	1,647	23.8	1,674	23.5	1,558	23.8	8,012	21.5	3,608	20.2
Mountain	25,118	25.0	2,350	22.9	2,733	25.3	2,615	26.8	13,035	25.8	4,385	21.8
Rainforest	6,714	5.6	817	6.3	836	6.5	706	5.9	3,590	5.7	765	3.3
Level of education (in ≥ 3 years)												
Illiterate (in ≥ 15 years)	1,003	1.0	NA		NA		7	0.1	254	0.7	742	4.0
Without schooling (in 3–14 years)	1,005	1.7	934	28.3	64	0.9	7	0.1	NA		NA	
Initial/Pre-school	3,049	5.4	2,213	70.6	765	12.1	2	0.0	20	0.0	49	0.3
Elementary	12,401	17.0	26	1.1	5,547	86.0	607	9.9	2,074	4.1	4,147	25.5
High School	19,792	35.2	NA		68	1.0	4,999	86.0	10,537	35.6	4,188	37.1
Technical/Higher	22,512	39.7	NA		NA		224	3.9	18,262	59.6	4,026	33.1
DK/DA	23	-					-		16	-	7	-
Marital status (in ≥ 18 years)												
Single	5,273	19.6	NA		NA		NA		4,739	23.0	534	5.0
Co-habitating	9,380	21.8	NA		NA		NA		8,831	26.0	549	3.9
Married	23,975	48.3	NA		NA		NA		15,147	44.0	8,828	66.3
Separated	2,126	4.9	NA		NA		NA		1,688	5.2	438	3.4
Divorced	265	0.7	NA		NA		NA		162	0.6	103	1.1
Widower	3,276	4.7	NA		NA		NA		578	1.2	2,698	20.3
DK/DA	27	-							18	-	9	-

NA: not applicable; DK/DA: does not know/does not answer

^a^ Percentages adjusted by expansion factor, percentage in totals per line and percentage of categories by column.

^b^ Metropolitan area with the highest population concentration.

The 48.9% of the total number of insured persons over 18 years of age are mainly employed and have mostly fixed contracts (46.5%). However, if we evaluate the situation of occupation in each one of the life cycles, we show that those over 60 years old are mostly pensioners (53.2%) and the group between 18 and 59 years old (57.5%). Additionally, the average monthly income of affiliates between 18 and 59 years old is between S/. 1,000 and S/. 1,500 (32.0%); while those over 60 years old receive on average less than S/. 850 (37%) ( Table 2 ).

**Table 2:** Socioeconomic characteristics of the insured population over 18 years old.

Variable	Total		Age groups			

			18–59		≥ 60	
		
	n	%^a^	n	%^a^	n	%^a^
Occupation situation	44,321	100.0	31,163	81.0	13,158	19.0
Domestic worker	98	0.3	79	0.3	19	0.2
Self-employed	4,980	9.4	3,385	9.1	1,595	10.4
Worker (includes day laborer, workman)	3,620	10.2	3,344	12.0	276	2.5
Employee	18,196	48.9	16,770	57.5	1,426	12.0
Employer	354	0.8	268	0.8	86	0.6
Student	293	1.1	293	1.4	0	0.0
Housewife	8,826	18.0	6,000	17.3	2,826	21.1
Pensioner	7,050	11.3	523	1.6	6,527	53.2
Unemployed	904	-	501	-	403	-
Afiliados con actividad laboral^b^	27,248	100.0	23,846	93.1	3,402	6.9
Type of contract^b^						
Permanent contract	10,750	35.5	9,495	35.3	1,255	38.5
Fixed time contract	10,454	46.5	10,086	49.0	368	15.4
Temporary Contract	6,033	18.0	4,256	15.7	1,777	46.1
DK/DA	11	-	9	-	2	-
Monthly income of insured people^b^	27,248	100.0	23,846	93.1	3,402	6.9
< 850	4,818	17.9	3,702	16.5	1,116	37.0
850–1,000	2,386	11.6	2,110	11.7	276	10.6
1,000–1,500	8,148	32.0	7,486	32.7	662	23.0
1,500–3,000	6,307	30.6	5,801	31.4	506	19.9
3,000–5,000	913	6.0	794	5.9	119	7.0
5,000–10,000	248	1.7	209	1.7	39	2.0
10,000+	29	0.2	18	0.1	11	0.5
DK/DA	4,399	-	3,726	-	673	-

DK/DA: does not know/does not answer

^a^ Percentages adjusted by expansion factor. Percentage in totals per line and percentage of categories per column.

^b^ Insured people > 18 years who reported work activity in the previous week or who have permanent employment. Amount in soles (1 USD = 3,269 PEN).

The dwellings are mostly independent (95.0%) and of similar proportions in the three regions of the country. The condition of housing tenure is essentially proper (68.1%); however, this proportion increases in the coast and rainforest (71.2% and 70.8% respectively) and decreases up to 59.7% in the mountains. The dwellings are built predominantly with noble material (88.7%) on the coast; but in the mountains and rainforest only 72.4% and 64.6%, respectively, of the homes of the members are built with noble material. Additionally, a non-negligible percentage of households of affiliates in the rainforest do not have access to public water and sewer network services (15.8% and 16.1% respectively); however, in all three regions there is access to electricity in more than 98% of homes ( Table 3 ).

**Table 3:** Access to basic services on the insured population dwellings according to country regions.

Variable	Total		Regiones del Perú					

			Costa		Mountain		Rainforest	
			
	n	%*	n	%*	n	%*	n	%*
Homes of the Insured population	21,645	100.0	10,599	67.2	8,832	27.0	2,214	5.8
Type of dwelling								
Independent house	10,347	95.0	10,347	96.1	8,274	91.8	2,166	98.3
Not independent	248	4.9	248	3.9	541	8.0	48	1.7
Others	4	0.1	4	0.0	17	0.2	0	0.0
Condition of housing tenure								
Rented	1,471	20.6	1,471	18.7	1,899	25.7	436	19.0
Owning home	8,290	68.1	8,290	71.2	6,028	59.7	1,584	70.8
Relinquished	834	11.3	834	10.1	900	14.6	193	10.2
Material of predominance of housing								
Noble material	8,703	82.9	8,703	88.7	5,768	72.4	1,350	64.6
No noble material	1,896	17.1	1,896	11.3	3,064	27.6	864	35.4
Access to water								
Public System:	10,405	97.8	10,405	98.5	8,705	99.2	1,811	84.2
No Public System:	194	2.2	194	1.5	127	0.8	403	15.8
Access to drain								
Public System:	10,318	97.0	10,318	97.6	8,633	98.4	1,799	83.9
No Public System:	281	3.0	281	2.4	199	1.6	415	16.1
Access to electricty								
Yes	10,482	99.0	10,482	99.0	8,751	99.2	2,187	98.3
No	117	1.0	117	1.0	81	0.8	27	1.7

* Percentages adjusted by expansion factor. Percentage in totals per line and percentage of categories per column.

In the insured population older than five years old, it was identified that 34.5% perform some type of sport or physical activity at least once a week; It emphasizes that in young stages, 42.9% (members between six and 11 years old) and 53.1% (members between 12 and 17 years old) perform physical activity, decreasing up to 32.9% in adults and scarcely to 19.8% in affiliates over 60 years old. However, the daily consumption of vegetables and fruits is a habitual behavior in the members; highlights the highest daily consumption of fruits that vegetables in children between six and 11 years old (86.1% and 77.5% respectively) and adolescents (84.2% and 78.8% respectively) ( Table 4 ).

**Table 4:** Lifestyles of the insured population, from 5 years old, according to age group.

Variable	Total		Age group							

			6–11		12–17		18–59		≥ 60	
				
	n	%*	n	%*	n	%*	n	%*	n	%*
Insured people ≥ 5 years	56,612	100.0	6,444	12.5	5,846	11.0	31,163	62.0	13,159	14.5
Do some sport or physical exercise at least once a week										
Yes	20,649	34.5	3,267	42.9	3,539	53.1	11,141	32.9	2,702	19.8
No	35,963	65.5	3,177	57.1	2,307	46.9	20,022	67.1	10,457	80.2
DK/DA	1,078	-		-		-		-		-
Consume vegetables daily										
Yes	45,632	79.3	5,062	77.5	4,685	78.8	25,121	79.4	10,764	81.0
No	10,980	20.7	1,382	22.5	1,161	21.2	6,042	20.6	2,395	19.0
Consume fruits daily										
Yes	45,708	81.6	5,505	86.1	4,855	84.2	24,590	79.9	10,758	83.0
No	10,904	18.4	939	13.9	991	15.8	6,573	20.1	2,401	17.0
Add salt to food										
Yes	4,219	6.3	320	3.4	492	6.8	2,661	7.2	746	4.8
No	52,393	93.7	6,124	96.6	5,354	93.2	28,502	92.8	12,413	95.2

DK/DA: does not know/does not answer

* Percentages adjusted by expansion factor. Percentage in totals per line and percentage of categories per column.

The working population (insured ≥ 18 years) mostly does not report having work accidents (95%), only in 3.8% of those who work has a health condition worsened by work activity. Additionally, 14.4% of the working population reports suffering from a disease or chronic health condition; however, these frequencies change by age group: thus, the population over 60 years old is the one that mostly suffers from a chronic illness (52.5%), while in the group of affiliates between 18 and 59 years old only 12% report suffering from any chronic condition. The 3.5% of affiliates report being diagnosed with diabetes, 7.1% of arterial hypertension, more than 4% of dyslipidemia (high cholesterol and triglycerides) and 0.5% of renal failure ( Table 5 ).

**Table 5:** Accidents, symptoms or illness during the last year according to age groups.

Variable	Total		Age group					

			6–17		18–59		≥ 60	
			
	n	%*	n	%*	n	%*	n	%*
Insured people	62,659	100.0	12,290	21.2	31,163	55.7	13,159	13.0
Occupational safety (in ≥ 18 years)	27,248	100.0	NA		23,846	93.1	3,402	6.9
Workplace accidents								
None	25,335	95.0	NA		22,199	94.9	3,136	95.4
1–2	1,405	4.0	NA		1,216	4.1	189	3.6
3–4	155	0.3	NA		133	0.3	22	0.3
5+	263	0.7	NA		231	0.7	32	0.7
DK/DA	90	-			67	-	23	-
Disease aggravated by work								
Yes	1,451	3.8	NA		1,255	3.7	196	4.2
No	25,700	96.2	NA		22,518	96.3	3,182	95.8
DK/DA	97	-			73	-	24	-
Have chronic illness or discomfort								
Yes	11,932	14.4	280	2.7	4,652	12.1	6,914	52.5
No	50,727	85.6	12,010	97.3	26,511	87.9	6,245	47.5
Have diabetes								
Yes	2,885	3.5	7	0.04	1,016	2.7	1,860	15.1
No	59,774	96.5	12,283	99.96	30,147	97.3	11,299	84.9
Have high cholesterol								
Yes	4,702	5.4	19	0.2	2,392	5.7	2,291	16.8
No	57,957	94.6	12,271	99.8	28,771	94.3	10,868	83.2
Have high triglyceride								
Yes	3,480	4.1	24	0.2	1,872	4.6	1,581	11.7
No	59,179	95.9	12,266	99.8	29,291	95.4	11,578	88.3
Have some heart disease								
Yes	1,802	2.0	58	0.4	506	1.2	1,204	9.2
No	60,857	98.0	12,232	99.6	30,657	98.8	11,955	90.8
Have high blood pressure								
Yes	6,606	7.1	7	0.04	2,029	4.7	4,568	34.3
No	56,053	92.9	12,283	99.96	29,134	95.3	8,591	65.7
Have kidney stones								
Yes	970	1.1	24	0.2	636	1.4	307	2.1
No	61,689	98.9	12,266	99.8	30,527	98.6	12,852	97.9
Have impaired kidney function								
Yes	412	0.5	14	0.2	176	0.4	219	1.7
No	62,247	99.5	12,276	99.8	30,987	99.6	12,940	98.3

NA: not applicable; DK/DA: does not know/does not answer

* Percentages adjusted by expansion factor. Percentage in totals per line and percentage of categories per column.

Medical attention was required from 35.9% of the members in the last three months, and 73.1% of them received medical attention; health care was provided mostly in EsSalud health centers (68.9%), 4.2% in health centers of the Ministry of Health, 15.9% in private clinics and 8.5% in pharmacies. Members who received medical care took less than a month to get their appointment (87.3%); the perception of severity of the disease by the patient is greater than that of the professional who attended them (35.2% *versus* 25.8%). The requirement for medical assistance is higher in the mountains (43.4%) and a scant 26% in the rainforest. Likewise, the proportion of members who require assistance and receive medical attention is lower in the rainforest and mountain range of the country (64.8% and 67.9% respectively) and the medical care of these patients is in pharmacies in a significant percentage of the members (16.7% in the mountains and 11.2% in the rainforest). Additionally, it was identified that patients have a perception of greater severity of their health compared to that reported by the attending physician; thus, we show that when asked about the degree of severity of their disease, 35.2% of patients consider it to be serious, while the professional who treated them considers it to be serious only at 25.8% ( Table 6 ).

**Table 6:** Access to health services during the last 3 months.

Variable	Total		Regions of Peru					

			Coast		Mountain		Rainforest	
			
	n	%*	n	%*	n	%*	n	%*
Insured people	62,659	100.0	30,827	69.4	25,118	25.0	6,714	5.6
Requirement for medical assistance								
Yes	25,926	35.9	12,100	33.9	11,877	43.4	1,949	26.0
No	36,733	64.1	18,727	66.1	13,241	56.6	4,765	74.0
Received medical attention	25,926	100.0	12,100	65.7	11,877	30.3	1,949	4.0
Yes	19,062	73.1	9,353	75.9	8,441	67.9	1,268	64.8
No	6,864	26.9	2,747	24.1	3,436	32.1	681	35.2
Care Center	19,062	100.0	9,353	68.2	8,441	28.2	1,268	3.6
EsSalud HC	13,294	68.9	6,784	71.7	5,584	61.1	926	76.0
MINSA HC	928	4.2	353	3.9	520	5.1	55	2.9
Hospital FFAA/NP	24	0.1	17	0.1	7	0.1	0	-
Private health service	2,551	15.9	1,407	16.3	1,004	15.9	140	9.0
Pharmacy	1,911	8.5	560	5.0	1,216	16.7	135	11.2
Others	354	2.4	232	3.0	110	1.2	12	0.9
Time to obtain appointments (months) in those who received medical attention	19,062	100.0	9,353	68.2	8,441	28.2	1,268	3.6
Less than a month	15,329	87.3	7,547	85.4	6,710	91.5	1,072	94.8
1	1,179	9.3	799	10.5	338	6.4	42	4.1
2	217	1.9	158	2.2	54	1.3	5	0.4
3–4	183	1.3	144	1.6	35	0.6	4	0.6
5+	40	0.2	25	0.3	14	0.2	1	0.1
DK/DA	2,114	-	680	-	1,290	-	144	-
Time to medical care (months) in those who received it	19,062	100.0	9,353	68.2	8,441	28.2	1,268	3.6
Less than a month	15,373	87.4	7,586	85.8	6,712	91.0	1,075	92.5
1	1,185	9.6	820	10.9	327	6.5	38	5.8
2	185	1.7	120	1.8	57	1.4	8	1.2
3–4	175	1.1	130	1.2	43	0.8	2	0.4
5+	30	0.2	17	0.3	12	0.3	1	0.1
DK/DA	2,114	-	680	-	1,290	-	144	-
Gravity of the illness according to the attending professional	19,062	100.0	9,353	68.2	8,441	28.2	1,268	3.6
Nothing serious	11,241	71.4	5,846	72.1	4,668	69.1	727	72.9
Serious	4,383	25.8	2,299	25.5	1,807	27.3	277	23.1
Very serious	521	2.8	242	2.4	228	3.7	51	4.0
DK/DA	2,917	-	966	-	1,738	-	213	-
Perception of the disease	19,062	100.0	9,353	68.2	8,441	28.2	1,268	3.6
Nothing serious	9,578	59.9	5,120	61.8	3,823	54.2	635	63.2
Serious	6,339	35.2	3,117	34.2	2,808	38.1	414	32.2
Very serious	987	4.9	420	4.0	492	7.6	75	4.7
DK/DA	2,158	-	696	-	1,318	-	144	-

DK/DA: does not know/does not answer; HC: health center; EsSalud: Social Health Insurance; MINSA: Ministry of Health; FFAA/NP: Armed Forces/National Police

* Percentages adjusted by expansion factor. Percentage in totals per line and percentage of categories per column.

## DISCUSSION

The ENSSA survey of 2015 is the first population survey conducted with a sample that covers the entire population of social security affiliates in Peru. This article describes the design detail and the methodology used, as well as the most relevant general results obtained from the survey data.

The results of this report show that EsSalud has a population of age groups relatively similar to the one reported nationally by the Demographic and Family Health Survey (ENDES) of the same year ^13^ . Additionally, this report shows that the economic income of the affiliates is above the average minimum remuneration ^14^ and they have access to basic water, sewage and light services in proportions similar to the general population reported by the ENDES ^13^ . There are few studies that show the level of physical activity in the Peruvian population; however, in 2009 and 2010, the National Center for Food and Nutrition (CENAN), using the sample from the National Household Survey, measured the level of physical activity in more than 53,000 subjects between 15 and 69 years old; their results show a low level of physical activity (25.4% in women and 18.4% in men) ^15^ . However, by 2015, the analysis of the data from our survey reported a higher percentage of physical activity in young members, reaching 53.1% in affiliates between 12 and 17 years old, with respect to the data reported by CENAN. It is important to highlight that our results show a change of these frequencies in the two poles of the life cycle, reaching almost 50% in childhood and adolescence and falling drastically to a low 20% in those over 60 years old. In the same way, our results show that 7.1% of affiliates report having high blood pressure and 3.5% diabetes, values very similar to those reported by the National Institute of Statistics in 2017 ^16^ . Access to health, education and public services are elements that allow to measure the development of a society and part of the domains to consider to measure equity in a population; thus, ENSSA survey has data that allow to evaluate access to health services from the determining factors point of view, both related to the consumer (economic, social, demographic), and those relating to the supply of services (type of) attention, level of care, care institution; the initial results we reported in this article show that the demand for health care in our affiliates is low, that is, at the time of the survey only the 35.9% reported any symptoms or discomfort, disease, relapse of disease or accident, and the 73.1% of these receive care, highlighting surprisingly that 10.9% served in pharmacies or other non-formal services of health, despite being a population with health coverage. It will be interesting that, with the data obtained in this survey, future studies can identify the barriers and factors related to the lack of access to health, considering even more that the ENSSA has data from a population affiliated with a health insurance.

Within the limitations of the study we can mention that the data were obtained by self-report. Although it is true that the self-reported data may include a reporting bias, this statement depends, in the case of health, on the objective existence of the disease, the subjective experience of the problem, and factors related to that report, such as the existence of a previous diagnosis made by a doctor, the subjective interpretation of the diagnosis and the recall bias. However, according to the methodology used (probabilistic nature of sampling, with random selection of households and individuals), this guarantees its representativeness and ability to inference of the results to the insured population.

The main strength of the ENSSA survey is that it includes information that does not exist in the EsSalud administrative records, such as demographic, social, employment and income characteristics, and others, allowing to obtain information on the insured people and the population that is part of their environment (home), characterizing its epidemiological framework and thus allowing the generation of evidence for future interventions of community type, and not only at level of companies. This data source is an opportunity for decision-makers at institutional and extra-institutional level, since having population evidence can generate health and management interventions that help strengthen the health of insured people, both at the preventive and community care level, as well as at the level of health facilities.
